# Guidelines for adapting cognitive stimulation therapy to other cultures

**DOI:** 10.2147/CIA.S61849

**Published:** 2014-06-26

**Authors:** Elisa Aguirre, Aimee Spector, Martin Orrell

**Affiliations:** 1 Division of Psychiatry, University College London, London, UK; 2 Research and Development Department, North East London Foundation Trust, Goodmayes Hospital, Essex, UK; 3 Clinical, Educational and Health Research Psychology Department, University College London, London, UK

**Keywords:** CST, culture, adaptation, mild to moderate dementia, dementia, FMAP

## Abstract

Cognitive stimulation therapy (CST) has been shown to be an useful and cost effective intervention that increases cognition and quality of life of people with mild to moderate dementia. It is increasing in popularity in the UK and worldwide, and a number of research teams have examined its effectiveness in other contexts and cultures. However, it is necessary to develop clear evidence-based guidelines for cultural modification of the intervention. This article describes a community-based developmental approach to adapt CST to different cultures, following the five phases of the formative method for adapting psychotherapy (FMAP), an approach that involves collaborating with service users as a first step to generate and support ideas for therapy adaptation. Examples based on clinical and practical experience are presented, along with suggestions for applying these changes in different cultural contexts.

## Introduction

**Figure 1:**
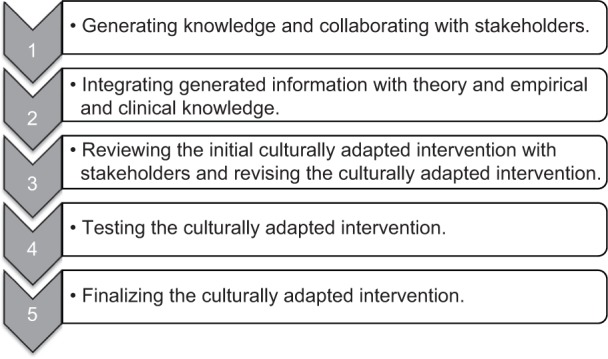
The five phases of the formative method for adapting psychotherapy.

There are different methods available to develop “cross-cultural adaptations”, the process that looks at both language (translation) and cultural adaptation issues in the process of preparing a questionnaire or therapy for use in another setting. To date, the majority of frameworks adapting therapies to different cultures take a “top-down” theoretical approach,^1^ proceeding from theoretical ideas of how best to culturally adapt programs. The purpose of the guidelines presented here is to describe a community based developmental approach in adapting cognitive stimulation therapy (CST)^2^ for people with mild to moderate dementia to different cultures. This method is based on the formative method for adapting psychotherapy (FMAP), a “bottom-up” approach that involves collaborating with service users as a first step to generate and support ideas for therapy adaptation.^1^ This approach consists of five phases (Figure 1) that target developing, testing, and reformulating of therapy modifications. This approach was also successfully used in development of the maintenance CST program.^3^

The World Alzheimer Report 2011,^4^ in a systematic review of psychosocial approaches for dementia care, concluded that cognitive stimulation had the “strongest evidence by far” for cognitive benefits in dementia. Cognitive stimulation for dementia has been defined as engagement of individuals (usually in a group) in a range of activities and discussions aimed at general enhancement of cognitive and social functioning.^5^ The best-evidenced and most widely used version of cognitive stimulation for dementia is CST.^2^ This brief, evidence-based group intervention was developed in the UK using a systematic approach based on theory and evidence from the three Cochrane reviews of reality orientation, reminiscence therapy, and validation therapy.^6^ The development of CST followed the Medical Research Council framework^7^ for the development of complex interventions. In phase two of the framework, the program was piloted,^8^ further modified, and extensively evaluated in a large and randomized controlled trial.^2^ The results showed that the CST group improved significantly in the main outcome measures of cognition and quality of life, that the intervention was cost effective, and that it compared favorably with the pharmaceutical intervention of cholinesterase inhibitors for Alzheimer’s disease in terms of numbers needed to treat.^2^,^9^ The basic CST program includes 14 sessions of 45 minutes each, occurring twice a week for 7 weeks. There is also a maintenance program available that includes 24 once-a-week sessions, each lasting for 45 minutes.^3^ Each session follows a theme or main activity (eg, current affairs, my life, word games) and is run by two facilitators. Each CST session begins with a warm-up activity, eg, participants play catch with a soft ball and introduce themselves; these are activities designed to encourage group interaction and increase alertness.

With any new psychosocial intervention one of the barriers to wide-scale international implementation is the need for suitable adaptation to other cultures. CST has been adapted to a number of different cultural groups. This paper articulates the experiences of translating and adapting CST to other cultures. In doing so, it provides a useful evidence-based guideline to translate and adapt the therapy to other cultures. Each of the phases of the FMAP model are described in the context of CST and can be tailored to meet the individual needs of different projects. CST adaptation is illustrated through examples of the modification of CST to several culturally diverse ethnic groups: Tanzanian and Nigerian communities, a Chinese community, a South Asian community, and a Japanese community. The cultural adaptation process can therefore be used as a guide for future research and practice.

## Methods and practical examples

### Phase one: generating knowledge and collaborating with stakeholders

The first step in adapting CST to a different culture is to decide which stakeholders to involve in the adaptation process and when to involve them. According to the FMAP, stakeholders may include (a) participants, (b) mainstream health and mental health care providers, (c) community-based organizations and agencies, (d) traditional and indigenous healers, and (e) spiritual and religious organizations.^1^ When adapting CST, it is recommended that the following stakeholders relevant to each culture are approached: (a) participants and family caregivers, (b) community health workers such as village health workers, (c) mental health care staff if available (psychiatrists, psychologists, social workers, therapists, and health care assistants), and (d) other older people with knowledge of relevant historical, cultural and religious issues. It is essential to include local health workers and staff, because these individuals typically have hands-on experience, knowledge, and expertise in working directly with people with dementia in their culture and context, and in providing services to them. Included stakeholders will be able to provide direct feedback on developing, adapting and improving CST. Eliciting participants’ feedback is also very important; however, it is recommended that this be delayed until phases four and five of the process.

A good way of consulting at this stage is through focus groups. Each focus group might consist of around four to six health workers with a range of clinical and working experiences. This will help facilitate both breadth and depth of discussions. The initial process of the focus group would include general discussions of cultural adaptation, and a review of the treatment manual and intervention as described in *Making a Difference: An Evidence-Based Group Programme to Offer Cognitive Stimulation Therapy (CST) to People With Dementia*^10^ and *Making a Difference 2: An Evidence-Based Group Programme to Offer Maintenance Cognitive Stimulation Therapy (CST) to People With Dementia*.^11^ Specifically, this could include participants’ impressions of CST, whether different aspects of CST would work in their particular culture, and to elicit feedback in relation to how best to modify CST for their community. It is especially helpful to involve diverse clinical disciplines, which possess different characteristics, biases, and perceptions relative to best practices. Diversity enables the collection of a range of feedback that can be used to produce a more ecologically-valid adaptation of the program.

A culturally specific example of the phase one process follows. In the South Asian adaptation of the program,^12^ an adaptation of most activities took place within each theme, using cultural and ethnic issues relevant to the Indian subcontinent. The program was run by two bilingual physicians in their first language, and a support worker. It was decided that the discussion of day, time, and place that routinely takes part at the beginning of each session, was going to be around events and issues relevant to localities in their countries of origin. Discussions included news of their local community, news around their own families and friends, or news from the daily TV soap operas they were following. Adapted activities for participants included local games such as a “carom board” for the number games session, familiar sounds from their past such as rickshaw and tonga (horse cart) for the sounds session, discussion around the immigration process, routes and difficulties for the orientation session, and famous historical personalities from the Indian subcontinent for the faces session.

Focus groups can also be complemented with individual interviews with other key practitioners in each culture who are not part of a particular setting. This provides an opportunity to exchange ideas, build a sense of community, and strengthen referral networks.

### Phase two: integrating generated information with theory and empirical and clinical knowledge

In this phase, the information generated from the community-based focus groups will be synthesized, and a new culturally adapted version of the manual can be written up. Focus group collaborations will help to reduce personal and clinician-specific biases, and collaboration with local health workers will help to ensure that cultural adaptations are grounded in cultural belief systems.

A culturally specific example of the phase two process follows. In the adaptation process of CST for Tanzania and Nigeria, the structure of sessions as described in the manuals had to be modified for logistical issues, as they would have not worked otherwise. Issues that influenced the structure included distances between villages, transport arrangements, carer commitments depending on the season, harvesting or planting crops, and access to villages during the rainy season.

Another consideration was the use of materials that had to be locally available and, most importantly, familiar to the group. It was also decided that the program location should be somewhat local, but where children could not observe the program process. Churches, mosques, or hospitals were also eliminated as locations, so that participants could feel comfortable regardless of their religion, and avoid feeling treated as sick.

### Phase three: review of the culturally adapted CST intervention by stakeholders, and further revision

After writing the culturally adapted CST manuals, it is recommended that a further focus group be conducted with the planned CST group facilitators. At this focus group, initial impressions of the adapted intervention, and feedback for improvement, will be elicited, paying special attention to the activities included in the revised and adapted manual. Feedback will be used to finalize manual content before implementation. The manual may then be written, and translated into the desired specific language. It is best practice to follow a standardized forward and back translation method with the back translation reviewed by the UK CST team. For example for the Japanese version, three experts were involved in the translation.^13^

Culturally specific examples of the phase three process follow. For the Tanzanian and Nigerian versions, three sessions were allocated to each member of the group for consideration and then discussed in the focus group. The main issues considered were: important local events for the centers and villages; how people become aware of current affairs (eg, sermon on the radio, TV, ceremonies); consideration of local materials for maps, food, sweets, games, or expansion to other areas in the country.

In adaptation of the program to South Asian groups, materials were tailored to South Asian cultures by including topics such as cricket due to its popularity in India and Pakistan. Other adapted sessions included preparing two different curry dishes for the “being creative” session, and sharing the dishes with the day center staff. An Urdu drama was shown and discussed as part of the “visual clips discussion” session. As most participants’ level of literacy was expected to be low (based on advice from family members), the program maximized the use of materials and equipment that incorporated visual (images) and auditory content that minimized the use of reading and writing.

### Phase four: testing the culturally adapted intervention

The developed and adapted CST manual should then be piloted in different settings in order to test the adapted program. This could involve running a full program in two carefully selected settings and using minimal outcomes to test its efficacy, such as cognitive and quality of life measures. Ideally, these would be community centers which normally provide services for people with dementia. Such locations would help ensure that the program is sustainable and that the developed intervention is feasible relative to frequency of sessions, number of participants, number of staff, duration of the sessions, transport, and financial limitations. In addition, getting other staff and managers within the selected community centers to participate in providing feedback from the pilot at this stage would help facilitate their support and adaptation of the CST program.

A culturally specific example of the phase four process follows. The Japanese version included a pilot study which revealed that it was necessary to modify the content of eight sessions that appeared unsuitable for the Japanese culture.^13^ Crossword puzzles in the session on word games were found not suitable in Japanese culture, so the alternative activity of “Shiritori” (a traditional Japanese word chain game) was substituted. Following analysis of results from the first pilot, the facilitators amended the content in eight of 14 sessions. These amendments were validated and confirmed by a second pilot in phase five as described below.

### Phase five: synthesizing stakeholder (participant and facilitator) feedback from pilot and finalizing the culturally adapted intervention

Facilitators and participants who take part in the culturally adapted pilot in phase four will be asked to take part in interviews or focus groups to elicit feedback regarding their experiences, what they found useful, what they did not like, and their recommendations for program improvement. Additional recommendations can be integrated within the adapted program manual. The adaptors of the CST program can use this information along with their own experiences to finalize the adapted CST manual.

A culturally specific example of the phase five process follows. In development of the Tanzanian and Nigerian adaptation it was noted that when asking questions of the participants, facilitators had to be culturally sensitive, and consider how much information the group would be comfortable sharing. An important element was to avoid asking for any personal information in the discussion, particularly names or numbers of children, unless participants elected to voluntarily share this information individually. Elements such as access to a comfortable table and chairs, refreshments, and bathroom were considered very important. Most participants expected a small gift after the sessions, so a gift, such as a confection, was provided at the end of every session; some participants enjoyed giving these to their grandchildren.

## Conclusion

These practical recommendations provide guidance on how to culturally adapt the content and structure of CST to make it more suitable to other cultures without compromising its effectiveness. The recommendations were based on clinical and practical experience plus evidence from a review of the most common frameworks that have been used to adapt therapy to other cultures. In particular, the guidelines have been grounded in the FMAP framework and its five phases, illustrating examples of currently modified programs. The available evidence from small studies of the adapted programs indicates that they are also of benefit.^12^,^13^

Although this manuscript describes guidelines for cultural adaptation of the CST program to other cultures, further studies will need to explore the cultural influences as a critical point. As previous literature shows, culture influences the understanding of dementia, the use of services, and the psychosocial experiences of family members.^14^–^16^ Therefore it is expected that interventions that have been developed with culturally and linguistically well-defined community-based methods may not consistently fit the standards, needs and expectations of the culture that influences beliefs and attitudes.^17^

Further research should pay attention to how the different culturally-explained definitions of old age and dementia in the different societal contexts where the intervention occurs, will shape and influence the effectiveness of this intervention in other cultures. Thus far there is little evidence-based research relative to how cultures vary in their availability and use of diagnostic procedures, and their use of different outcome measures. Further studies will need to explore and address these deficiencies in order to better understand the effectiveness of these adapted interventions. Finally, issues of empowerment and collaboration with service users are echoed within this article.
